# Hemodynamic evaluation of patients with Moyamoya Angiopathy: comparison of resting-state fMRI to breath-hold fMRI and [^15^O]water PET

**DOI:** 10.1007/s00234-021-02814-8

**Published:** 2021-09-27

**Authors:** Leonie Zerweck, Constantin Roder, Till-Karsten Hauser, Johannes Thurow, Annerose Mengel, Marcos Tatagiba, Nadia Khan, Philipp T. Meyer, Ulrike Ernemann, Uwe Klose

**Affiliations:** 1 Department of Diagnostic and Interventional Neuroradiology, University Hospital Tuebingen, Tübingen, Germany; 2 Department of Neurosurgery, University Hospital Tuebingen, Tübingen, Germany; 3 Department of Nuclear Medicine, Medical Center – University of Freiburg, Faculty of Medicine, University of Freiburg, Freiburg, Germany; 4 Department of Neurology and Stroke, University Hospital Tuebingen, Tübingen, Germany; 5 Moyamoya Center, University Children’s Hospital Zurich, Zurich, Switzerland

**Keywords:** Cerebrovascular reactivity, Resting-state fMRI, Breath-hold fMRI, [^15^O]water PET, Moyamoya Angiopathy

## Abstract

**Purpose:**

Patients with Moyamoya Angiopathy (MMA) require hemodynamic evaluation to assess the risk of stroke. Assessment of cerebral blood flow with [^15^O]water PET and acetazolamide challenge is the diagnostic standard for the evaluation of the cerebral perfusion reserve (CPR). Estimation of the cerebrovascular reactivity (CVR) by use of breath-hold-triggered fMRI (bh-fMRI) as an index of CPR has been proposed as a reliable and more readily available approach. Recent findings suggest the use of resting-state fMRI (rs-fMRI) which requires minimum patient compliance. The aim of this study was to compare rs-fMRI to bh-fMRI and [^15^O]water PET in patients with MMA.

**Methods:**

Patients with MMA underwent rs-fMRI and bh-fMRI in the same MRI session. Maps of the CVR gained by both modalities were compared retrospectively by calculating the correlation between the mean CVR of 12 volumes of interest. Additionally, the rs-maps of a subgroup of patients were compared to CPR-maps gained by [^15^O]water PET.

**Results:**

The comparison of the rs-maps and the bh-maps of 24 patients revealed a good correlation (Pearson’s *r* = 0.71 ± 0.13; preoperative patients: Pearson’s *r* = 0.71 ± 0.17; postoperative patients: Pearson’s *r* = 0.71 ± 0.11). The comparison of 7 rs-fMRI data sets to the corresponding [^15^O]water PET data sets also revealed a high level of agreement (Pearson’s *r* = 0.80 ± 0.19).

**Conclusion:**

The present analysis indicates that rs-fMRI might be a promising non-invasive method with almost no patient cooperation needed to evaluate the CVR. Further prospective studies are required.

## Introduction

Moyamoya Angiopathy (MMA) is a progressive steno-occlusive arteriopathy of terminal parts of the internal carotid arteries and the circle of Willis [1] resulting in reactive collateralization from new vessels. The most common symptoms are transient ischemic attacks, ischemic strokes, headache, and epilepsy [2–4]. Since no causal treatment to stop the steno-occlusive disease exists, the therapeutic approach consists of surgical revascularization, for example, by bypass surgery between the superficial temporal artery and the middle cerebral artery [5]. If a reduced cerebral perfusion reserve capacity is seen on functional imaging, patients have an increased risk of stroke. Therefore, for the indication of neurosurgical revascularization cerebral perfusion reserve (CPR) must be measured by functional perfusion imaging [6, 7].

Today’s investigative standards of the CPR include nuclear medicine imaging techniques such as [^15^O]water PET with acetazolamide (ACZ) challenge [8]. Disadvantages are high cost and limited availability due to the requirement of short-lived radiopharmaceuticals. In addition, absolute quantification of cerebral blood flow requires invasive arterial blood sampling, which, however, may be circumvented by semi-quantitative, non-invasive approaches [9, 10]. Although very rare, side effects of the application of ACZ may occur [11]. Functional blood-oxygen-level-dependent (BOLD) fMRI with hypercapnic stimulation provides a more widely available but comparable alternative by measuring the cerebrovascular reactivity (CVR), which is defined as the change in cerebral perfusion in response to a vasodilatory stimulus as an index for the CPR [10]. CO_2_ is a potent vasodilatory stimulus that leads to increased perfusion in brain tissue with unimpacted CVR [12]. Since the cerebral metabolic rate of oxygen remains constant, the increased perfusion results in an altered ratio of diamagnetic oxy-hemoglobin to paramagnetic deoxy-hemoglobin and causes significant BOLD signal changes [13]. Hypercapnia-triggered fMRI does not require the application of radiopharmaceuticals and is therefore less restricted by contraindications (e.g., pregnancy). Hypercapnia can be achieved by the inhalation of CO_2_-enriched gas [12, 14–19] or by performing short breath-hold periods [10, 12, 20–23]. The inhalation of CO_2_-enriched gas mixtures requires experience in handling and monitoring [24] and an MR-compatible gas delivery apparatus [25] that might be not available in every hospital. Breath-holding (bh) is an easily implementable method to achieve hypercapnia and does not require any additional equipment [11, 26, 27], but relies on the patients’ cooperation [11, 12].

Recent findings suggest that resting-state fMRI (rs-fMRI) might be a promising method to estimate the CVR [24, 25, 28–31]. One method uses low-frequency physiological CO_2_ oscillations during normal breathing as an intrinsic vasoactive stimulus to estimate the CVR [24, 28]. These small fluctuations in arterial CO_2_ are expected in a frequency range of 0.05 Hz and below [24, 32–34] and attributed to fluctuations in breathing depth, breathing rate, and delayed chemoreflex feedback [33, 35]. The rs-fMRI approach attempts to investigate the BOLD signal response to these global intrinsic CO_2_ variations attributed to natural variations in respiration. One methodology is to extract BOLD MRI signal components attributed to CO_2_ fluctuations by means of a suitable frequency band-pass filter and to use the BOLD signal time course of a brain region in which a physiological CVR is expected as a reference region for voxel-wise regression analysis [24, 28]. In this context, rs-fMRI is not used to analyze functional connectivity, such as the default mode network, as in other clinical settings. Rather, the aim is to detect different respiration-induced BOLD signals in brain regions with impaired CVR than in healthy reference regions during natural breathing. Recently, Liu et al. showed that rs-fMRI leads to comparable results as hypercapnia-triggered fMRI by use of CO_2_-enriched gas inhalation [24].

Breath-hold-triggered BOLD fMRI (bh-fMRI) has been shown to lead to reliable and comparable hemodynamic evaluation of patients with MMA to the diagnostic standard [^15^O]water PET with ACZ challenge [10, 23]. In this work we aimed to estimate the CVR in patients with MMA by use of rs-fMRI and compared the results to bh-fMRI and to the assessment of the CPR yielded by [^15^O]water PET with ACZ challenge.

## Methods

A retrospective analysis of 27 rs-fMRI and 27 bh-fMRI data sets of 26 patients with MMA was performed. Both fMRI examinations were performed in the same session during routine clinical MRI scans to evaluate cerebral hemodynamics. Main inclusion criteria were angiographically proven MMA with reduced CVR in at least one vascular territory (bh-triggered BOLD signal increase reduced by at least 30% compared to the signal increase of a cerebellar reference region, see below) and no secondary cerebral disease. The data sets of two patients were excluded because no impairment of the CVR was observed. Morphological MR imaging showed lesions in 14 of the patients with MMA. The data sets of these patients were evaluated in a subgroup and compared to the subgroup of data sets with unremarkable morphological imaging. None of the patients included in the study presented with seizures at the time of examination. Data sets of 14 patients before and 11 patients after neurosurgical revascularization were included in this study and evaluated combined and in subgroups. One patient was examined twice, once before and the second time 4 months later after surgical revascularization. General patient data can be seen in Tab. 1. Additionally, in a subgroup of seven patients the rs-fMRI data sets were compared to [^15^O]water PET with ACZ challenge if these data sets were available. The time period between the MR and the [^15^O]water PET examination was up to 3 months. The study was approved by the local Ethics Committee.

**Table 1:** Overview of General patient data

General patient data	
Mean age (range)	48 (9–75)
Female:male ratio	16:9
Bilateral Moyamoya disease	20
Unilateral Moyamoya Angiopathy	5
rs-fMRI data sets	25
bh-fMRI data sets	25
MR imaging before revascularization	14
MR imaging after revascularization	11
MR imaging with lesions	14
MR imaging without lesions	11
PET data sets available	7

### MRI data acquisition

All MR images were acquired on a 3-T MR Scanner (Magnetom Skyra, Siemens, Erlangen, Germany). During the examination, subjects were positioned supine on the scanner table. A standard 20-channel head coil was used for imaging. The fMRI data was measured by means of T2*-weighted echo-planar sequences with the following parameters: resting-state fMRI: TR = 2000 ms, TE = 35 ms, matrix 64 × 64, 3-mm slice thickness, 34 slices in interleaved ascending order, FOV = 190 mm, resolution 3.0 × 3.0 × 3.0, echo spacing: 0.58 ms, TA 5:12 min, 153 measurements. Breath-hold fMRI: TR = 3000 ms, TE = 36 ms, matrix 96 × 96, 3-mm slice thickness, 34 slices in interleaved ascending order, FOV = 245 mm, resolution 2.6 × 2.6 × 3.0, echo spacing: 0.58 ms, TA 6:53 min, 135 measurements.

During the rs-task the patients were instructed to lie still and close their eyes without performing any task.

The bh-task involved 60 s of normal breathing, followed by 5 repetitive cycles, each consisting of 9-s end-expiratory breath-hold periods and 60 s of regular breathing. The respiratory instructions were presented visually via a wall-mounted display by use of mirror fixed to the head coil. We used Presentation V20.1 (Neurobehavioral Systems, Berkeley, CA, USA) to present scanner triggered stimuli.

### MRI data pre-processing

**Fig. 1:**
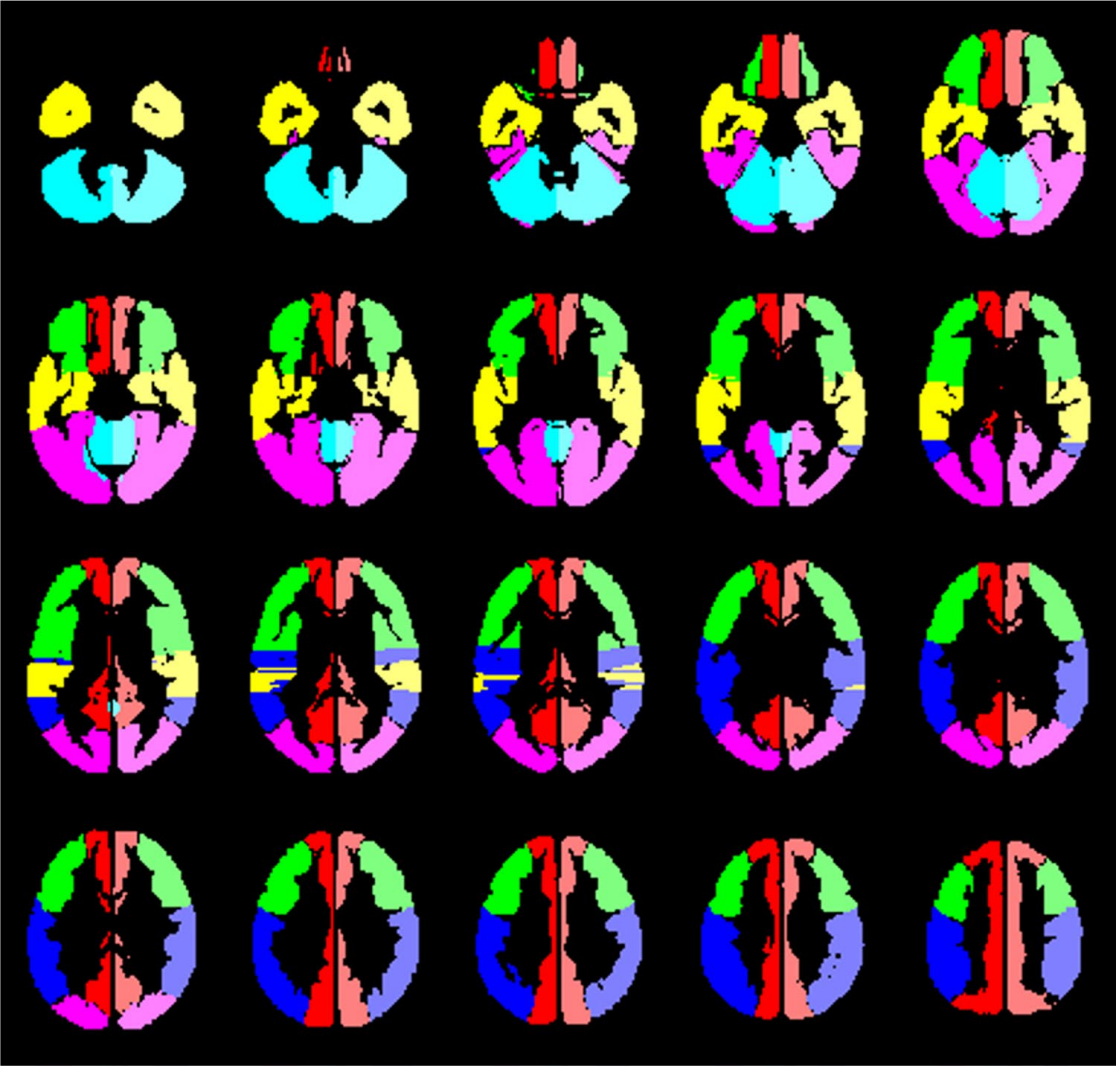
The 12 evaluated volumes of interest based on the vascular territory of the anterior cerebral artery (red), the frontal (green), temporal (yellow), and parietal (blue) territories of the middle cerebral artery, the territory of the posterior cerebral artery (pink), and mainly the cerebellum/brainstem (turquoise), which were evaluated separately for both hemispheres

The MRI data was pre-processed using Statistical Parameter Mapping (SPM12) (https://www.fil.ion.ucl.ac.uk/spm/) running on MATLAB (R2018b (The MathWorks, Inc., Natick, MA; http://www.mathworks.com)). The DICOM images were converted to analyze format in NIfTI (Neuroimaging Informatics Technology Initiative) and then realigned to correct subjects’ head movement, normalized to standard MNI space, segmented into 12 templates [36] (see Fig. 1) to enable good comparability between the rs- and the bh-data, and spatially smoothed by a Gaussian kernel of 12-mm FWHM. The bh-data was slice-timing corrected to equalize the time of image acquisition. The rs-images were not slice-timing corrected because the frequency of resting-state BOLD-signal fluctuations attributed to low-frequency CO_2_ oscillations is expected to be much lower [24, 32–34] than the MR sample frequency in this study (TR = 2 s). The rs-data was temporally filtered with a band-pass filter of 0.02 to 0.04 Hz, because previous findings of Liu et al. suggest that global resting-state BOLD signal fluctuations within this frequency range correlate best with natural end-tidal CO_2_ fluctuations [24]. All further processing of the data was performed by using in-house scripts programmed in MATLAB.

### Analysis of rs-fMRI data

We used the above-mentioned template of mainly the cerebellum/brainstem (see Fig. 1) and calculated the mean signal time course of this reference region to yield a reference signal time course for voxel-wise correlation analysis. We performed a cross-correlation analysis in which the correlation coefficients between the cerebellar reference time course and the individual voxels’ signal time courses were calculated. The values of the correlation coefficients were displayed as overlays on the normalized standard brains. In exemplary maps generated in this manner, color-coding was applied, with high correlation coefficients represented by warm colors and low correlation coefficients by cold colors. For an individual color-coding, the histogram of all voxels within the cerebellar reference volume was calculated and the 20th percentile of the cerebellar histogram was determined. All voxels within the brain with a correlation coefficient exceeding the 20th percentile of the histogram were colored red. The threshold values of the further color-coding resulted from a gradual subtraction of 0.2 from this individual cerebellar threshold value.

### Analysis of bh-fMRI data

**Fig. 2:**
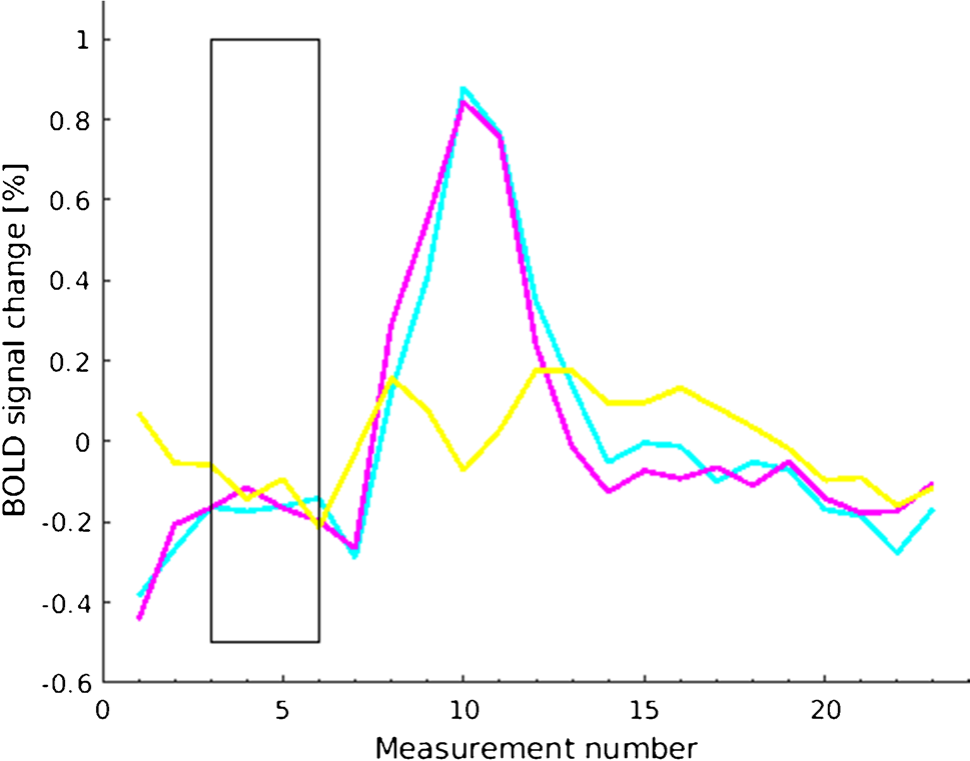
Exemplary BOLD signal time courses of one patient’s breath-hold measurement averaged over the 5 cycles. The rectangle marks the breath-hold period. Shown is a reference region (right cerebellum, turquoise), a region with physiological CVR (territory of the right posterior cerebral artery, pink), and a region with reduced CVR (temporal territory of the right middle cerebral artery, yellow)

The template of mainly the cerebellum was used to calculate the signal time course of each of the five repetitive cycles. This served to check the compliance of the patients in performing the breath-hold periods. For each voxel, the signal time course of the five cycles was calculated and averaged (Fig. 2). The relative BOLD signal change was calculated to adjust for variability in the baseline signal intensity of the echo planar imaging sequence. The signal time course of the cerebellum also served as reference region to manually select the start and the end point of the breath-hold related BOLD response in each patient. For a more detailed description of the procedure and exemplary depictions of the signal time courses we refer to the study of Hauser et al. [10]. Mean relative signal change during the cerebellar BOLD response period was calculated voxel-wise and displayed as overlays on the normalized standard brains. In exemplary maps generated in this manner, color-coding was applied with high values assigned to warm colors and low values to cold colors to enable optimal comparability with the rs-maps. Analogous to the color-coding of the rs-maps, the 20th percentile of the cerebellar histogram was determined as threshold value for the color red. The threshold values of the other colors were calculated by gradually subtracting 10% from this value, resulting in the thresholds 90%, 80%, 70%, etc. of the 20th percentile of the cerebellar histogram.

### [^15^O]water PET with ACZ challenge: data acquisition, processing, and analysis

PET scans (in *n* = 7 patients; *n* = 6 preoperative, *n* = 1 postoperative) were acquired on a Philips Vereos digital PET/CT system (Philips, The Netherlands). We did not absolutely quantify the regional cerebral blood flow (CBF; in terms of ml/min/100 g brain tissue) by continuous arterial blood sampling and kinetic modeling. Instead we used a simplified method to estimate cerebral perfusion reserve (CPR) as proposed by Arigoni et al. [9] and as recently described [10].

After a low-dose CT for attenuation correction, four 4-min scans were acquired per patient. The head position was gently restrained by a tape, carefully monitored, and, if necessary, corrected throughout the procedure by means of reference skin marks and the scanner laser beam. Scans before and after ACZ injection were done in duplicate (10-min interval between [^15^O]water injections to allow for sufficient decay) under resting conditions (i.e., eyes open and ears unplugged at normal ambient light and noise). A standard dose of 1000 mg ACZ (except in one patient who received 800 mg because of low body weight of 54 kg) was dissolved in 10 ml water for injection and injected immediately after the end of the 2nd baseline scan as an infusion over 5 min. The 3rd PET scan was started 10 min (5 min) after the start (end) of the ACZ infusion. A standard dose of 300 MBq [^15^O]water (10-ml volume) was administered at the start of each PET scan as a bolus over 3 s into a cubital vein. PET data sets were reconstructed by using a line-of-response time-of-flight ordered subset 3D iterative reconstruction algorithm employing spherically symmetric basis functions (so-called BLOB-OS-TF reconstruction; number of iterations = 3, number of subsets = 11, resulting voxel size = 2.0 × 2.0 × 2.0 mm^3^) yielding a dynamic sequence of 30 frames (18 × 5 s, 9 × 10 s, and 3 × 20 s) per scan. All datasets were fully corrected (attenuation, scatter, randoms, normalization, and calibration) during iterative reconstruction.

For simplified voxel-wise calculation of CPR maps, all scans of each subject were corrected for motion and integrated over 60 s after arrival of the tracer in the individual patient’s brain [9, 10]. The start time of integration was determined by analyzing the time-activity curve with a whole-brain region of interest (35% of maximum isodensity contour) covering a 5-cm slab at the level of the basal ganglia. Voxel-wise estimate of CPR (CPR map) were calculated using the SISCOM methodology [37]. In brief, the two integral scans before and after ACZ were averaged and then masked using a threshold of 18% of the image maximum (whole-brain segmentation). Voxel-wise signal change (%) from baseline status to the status after ACZ administration was calculated and the resulting parametric map was smoothed (Gaussian filter, 12-mm FWHM) and masked again (see above), yielding the desired CPR map. A customized “UCLA 2” color-scale was used for display of the CPR maps (thresholded symmetrically around zero with the zero transition being marked by three black bins within the 256-bin color-scale). The commercial software packages PMOD (version 3.7; PMOD Technologies LLC, Switzerland) and MATLAB (The MathWorks, Inc., Natick, MA, USA), as well as the freely available Statistical Parametric Mapping (SPM 12, http://www.fil.ion.ucl.ac.uk/spm/) were used for the aforementioned analyses.

### Comparative analysis of rs-fMRI with bh-fMRI

We calculated the mean CVR values of the above mentioned 12 VOIs of each patient (rs-fMRI: correlation coefficients, bh-fMRI: mean relative signal change). The correlation between the normalized rs- and bh-maps was calculated with Pearson’s correlation coefficient *r*, comparing the mean CVR of the 12 VOIs. For each test *p* < 0.05 was considered statistically significant.

### Comparative analysis of rs-fMRI with [^15^O]water PET

Analogous to the comparison between rs-fMRI and bh-fMRI, a comparative analysis of rs-fMRI with [^15^O]water PET was performed by calculating the mean CVR and CPR values of the 12 VOIs (rs-fMRI: correlation coefficients, [^15^O]water PET: mean blood flow changes (%) after ACZ stimulation). The correlation between the normalized rs- and PET-maps was calculated with Pearson’s correlation coefficient *r* comparing the mean CVR/CPR of the 12 VOIs.

## Results

In total, 25 rs-fMRI data sets of 24 patients with MMA were analyzed and compared to the corresponding bh-fMRI data sets and seven of the rs-fMRI data sets were compared to the corresponding [^15^O]water PET data sets. All imaging data sets were of good quality and no data set had to be excluded from the evaluation. Visual analysis of the cerebellar signal time courses revealed that the breath-hold tasks were accurately performed in all patients and that all data sets could be included in the analysis.

### Comparison of rs-fMRI and bh-fMRI

**Fig. 3:**
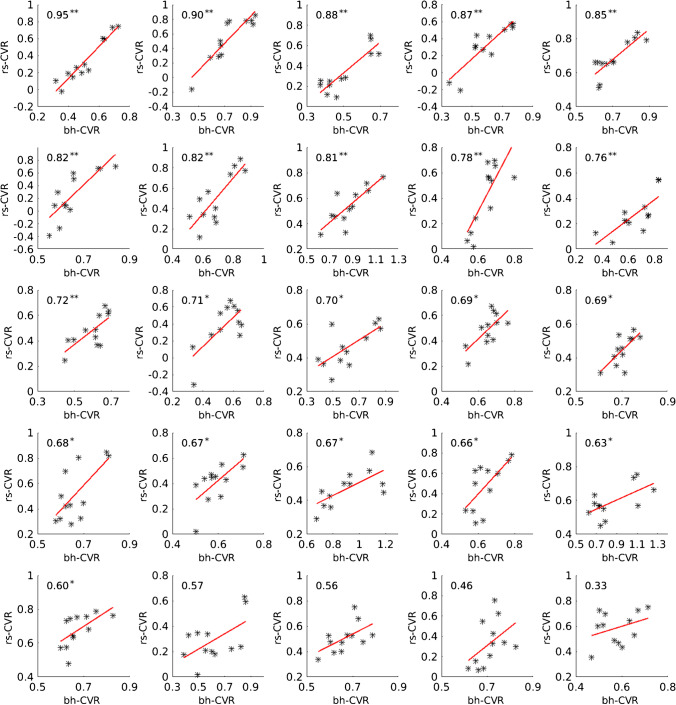
Scatter plots showing the correlation between rs-fMRI CVR (correlation coefficients) and bh-fMRI CVR (mean relative signal change) of 12 VOIs of each patient’s measurement sorted by Pearson’s correlation coefficient in descending order. Two asterisks (**) mark significant correlation at *p* < 0.01. One asterisk (*) marks significant correlation at *p* < 0.05

**Fig. 4:**
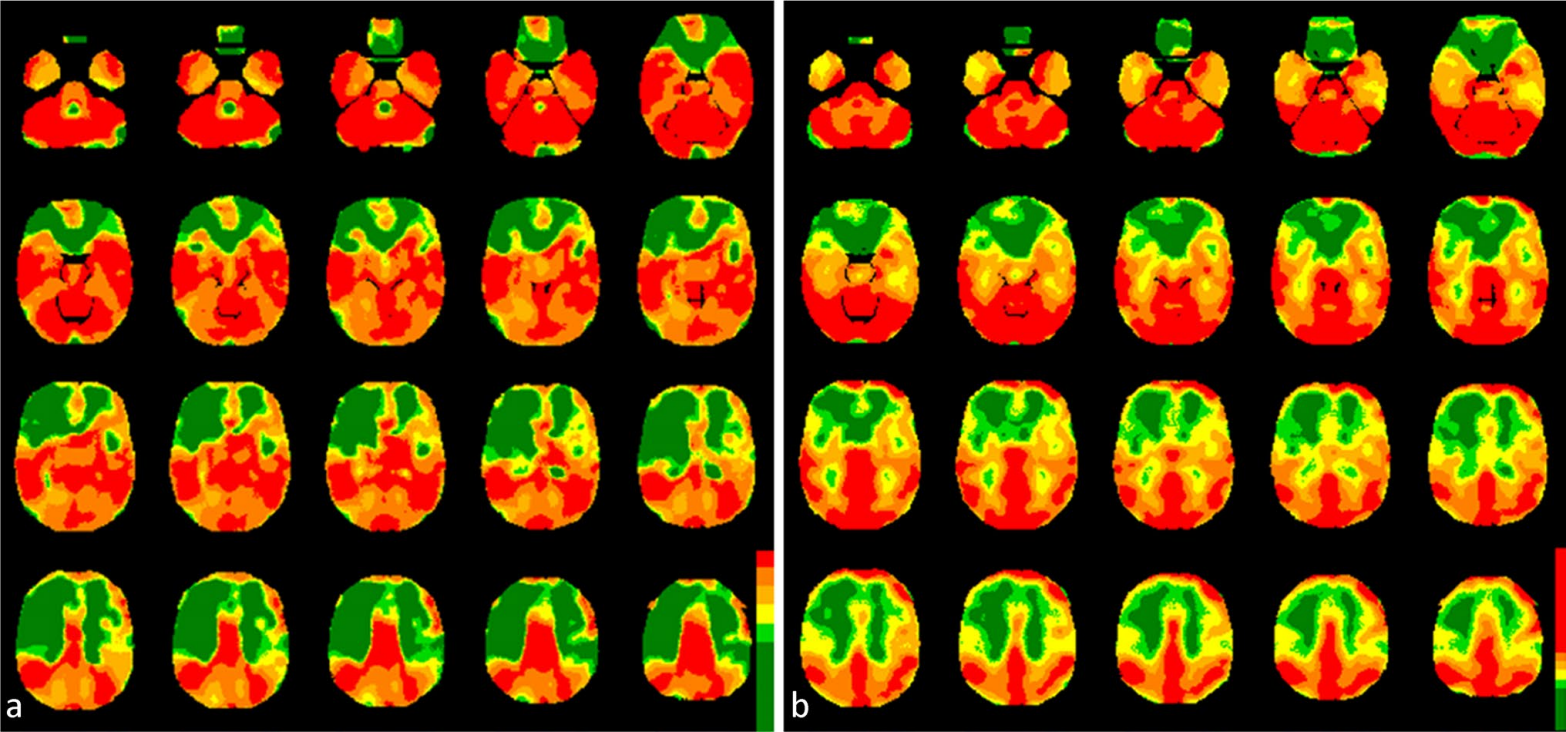
CVR-maps of one patient (*r* = 0.90) gained by rs-fMRI (**a**) and bh-fMRI (**b**). The CVR values (rs-fMRI: correlation coefficients, bh-fMRI: mean relative signal change) are presented as overlays on normalized anatomical MRI images. Color-coding was applied with high CVR resembled by warm and low CVR by cold colors. The color-coding of both modalities was applied in standardized gradations using thresholds based on the individual 20th percentile of the cerebellar histograms (see “Methods” section). Both the CVR-maps gained by rs-fMRI and by bh-fMRI indicate reduced CVR in the territories of the anterior and the middle cerebral arteries whereat the right hemisphere is more affected than the left

The comparison between the CVR in the investigated VOIs gained by rs-fMRI and bh-fMRI revealed a significant correlation in 21 of 25 data sets (Fig. 3). High correlation coefficients (maximum 0.95) were measured in most patients. Four patients showed low, non-significant correlation coefficients (minimum 0.35). In three of these patients, significant correlation coefficients were detectable when evaluating only 50% of the slices (centrally located in the standardized MNI space). In one patient differences in the signal time courses of the VOIs with physiological and reduced CVR were detectable only during a short time period at the end of the acquisition time. The mean Pearson’s correlation coefficient was 0.71 ± 0.13. A strong correlation could be detected in the preoperative subgroup (mean Pearson’s correlation coefficient 0.71 ± 0.17) as well as in the postoperative subgroup (mean Pearson’s correlation coefficient 0.71 ± 0.11). Comparing the subgroups of patients with lesions (mean Pearson’s correlation coefficient 0.71 ± 0.12) and without lesions (mean Pearson’s correlation coefficient 0.72 ± 0.18), no significant differences in the correlation between rs-fMRI and bh-fMRI were found. Pearson’s correlation coefficient in the subgroup of seven patients with PET data sets available was 0.79 ± 0.21. Exemplary color-coded CVR maps achieved by rs-fMRI and bh-fMRI can be seen in Fig. 4.

### Comparison of rs-fMRI and [^15^O]water PET

**Fig. 5:**
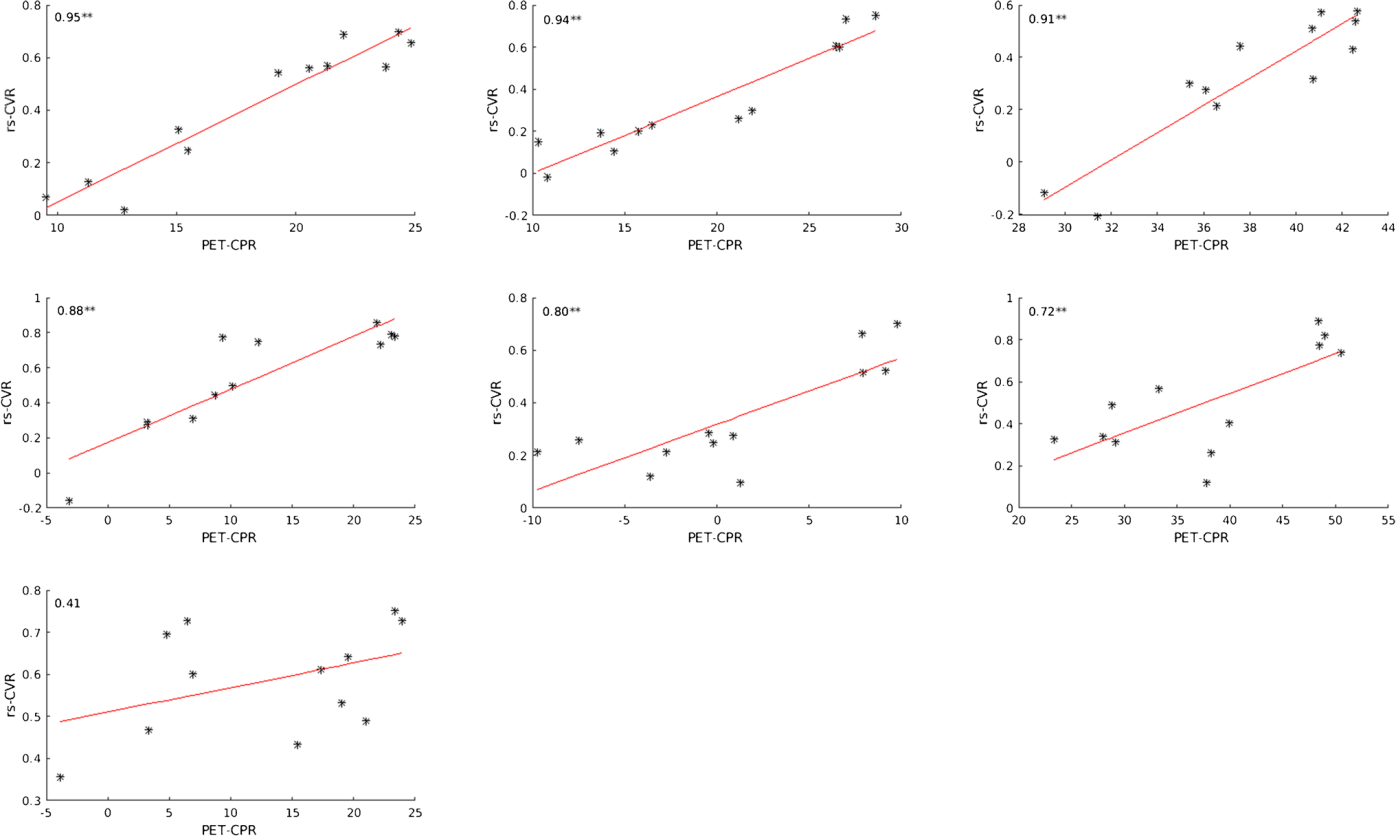
Scatter plots showing the correlation between rs-fMRI CVR (correlation coefficients) and [^15^O]water PET CPR (mean cerebral blood flow changes (%) after ACZ stimulation) of 12 VOIs of each patient’s measurement sorted by Pearson’s correlation coefficient in descending order. Two asterisks (**) mark significant correlation at *p* < 0.01. One asterisk (*) marks significant correlation at *p* < 0.05

**Fig. 6:**
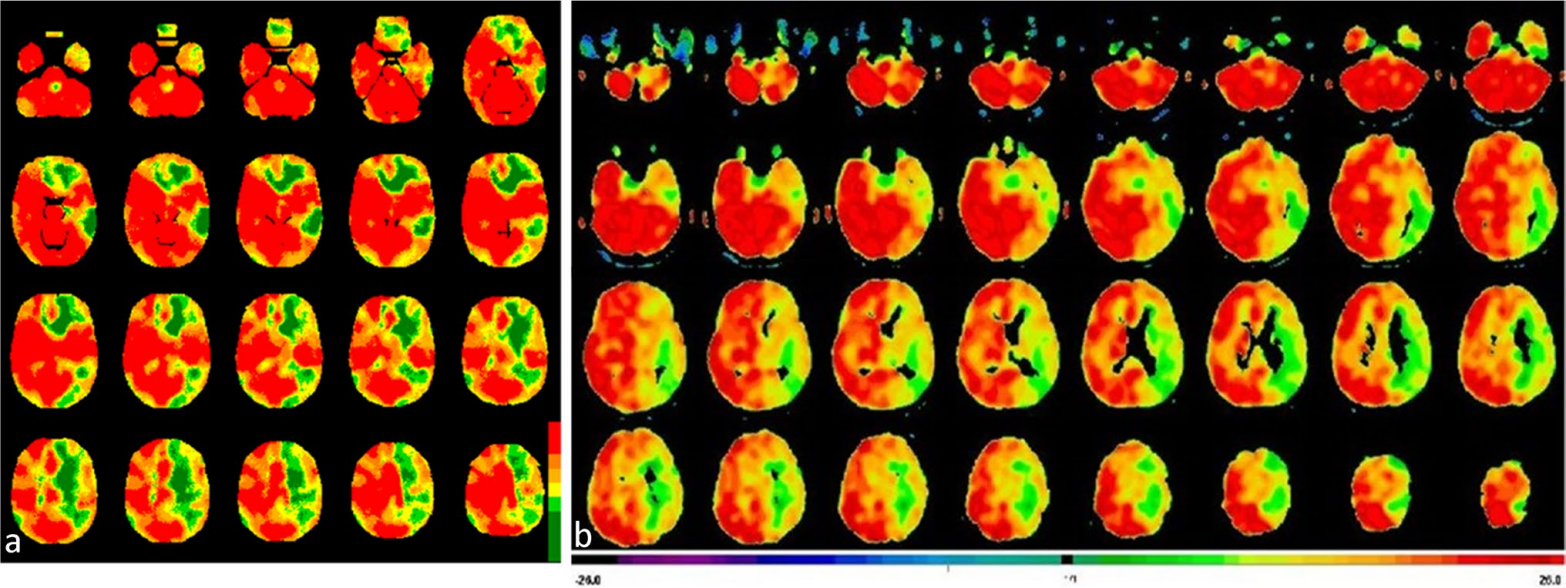
CVR-maps of one patient (*r* = 0.95) gained by rs-fMRI (**a**) and the corresponding CPR-maps gained by [^15^O]water PET with ACZ challenge (**b**). Color-coding was applied with high CVR/CPR resembled by warm and low CVR/CPR by cold colors. The rs-fMRI maps represent voxel-wise correlation coefficients to the cerebellum. All voxels exceeding the 20th percentile of the cerebellar histogram (correlation coefficient = 0.77) are colored red. The threshold values of the remaining colors result from a gradual subtraction of 0.2. Voxel-wise cerebral blood flow changes (%) after ACZ stimulation as provided by [^15^O]water PET are color-coded (modified “UCLA 2” color-scale, see “Methods” section). The maps of both modalities indicate perfusion deficits in the left hemisphere

In total, seven rs-fMRI data sets and the corresponding [^15^O]water PET data sets were compared. A strong and significant correlation (maximum 0.95) could be detected in six of seven patients (mean Pearson’s correlation coefficient 0.80 ± 0.19 in all *n* = 7) (see Fig. 5). Color-coded maps of one case can be seen in Fig. 6. The rs-fMRI data set with weak correlation to [^15^O]water PET (0.41) (see Fig. 5, patient 7) also correlated weakly to the corresponding bh-fMRI data set (see Fig. 3, patient 25). Compared to [^15^O]water PET and to bh-fMRI, the CVR deficits in the affected vascular territories of this patient could not be adequately represented by means of rs-fMRI. An analysis of the resting-state BOLD signal time courses of this patient revealed that differences in the signal time courses of the VOIs with physiological and reduced CVR were detectable only during a time period at the end of the data acquisition. In this patient, the amplitude of the BOLD signal changes was relatively high during the whole time period and the signal time courses of all regions showed a high similarity. Since no respiratory monitoring was performed, we can only assume, but it is possible that the patient showed an atypical respiratory pattern, so that the selected frequency band-pass filter was inappropriate in this patient. An analysis of the rs-fMRI data sets of this patient without frequency filtering showed a higher correlation to the corresponding [^15^O]water PET data sets (Pearson’s correlation coefficient 0.55).

## Discussion

In patients with Moyamoya Angiopathy functional perfusion imaging is required to indicate the need for neurosurgical revascularization [6, 7]. Nuclear medicine imaging techniques such as [^15^O]water PET with ACZ challenge are established diagnostic methods for the assessment of the remaining cerebral reserve capacity and considered as the diagnostic standard [8]. In recent years, non-invasive MR-based diagnostic methods have gained in importance for CVR estimation [8, 11, 12, 18–20, 38–40]. The vasodilatory stimulus can be hypercapnia achieved by breath-holding [11, 12]. Recent studies have demonstrated not only the strong correlation between bh-fMRI and [^15^O]water PET [10], but also the high longitudinal reproducibility of the procedure in the hemodynamic evaluation of patients with MMA [23]. However, a disadvantage is the dependence on the patients’ cooperation. Latest approaches attempted to exploit natural low-frequency fluctuations in arterial CO_2_ during normal breathing to estimate the CVR [24, 25, 28, 31, 41]. In this study we compared the new rs-fMRI approach to breath-hold-triggered fMRI in patients with MMA. Additionally, in a subgroup of seven patients, rs-fMRI was compared to [^15^O]water PET.

In the present analysis of patients with MMA the estimation of the CVR by use of rs-fMRI revealed a good correlation with bh-fMRI (*r* = 0.71 ± 0.13). Both the comparison of the subgroup of preoperative (*r* = 0.71 ± 0.17) and postoperative patients (*r* = 0.71 ± 0.11) correlated strongly. The correlation of the rs-fMRI data sets to the bh-fMRI data sets was independent of the presence (*r* = 0.71 ± 0.12) or absence of lesions (*r* = 0.72 ± 0.18) in the patients’ morphological imaging. The comparison of the CVR maps gained by rs-fMRI to the CPR maps gained by [^15^O]water PET revealed a strong agreement (*r* = 0.80 ± 0.19), which was comparable to the correlation between rs-fMRI and bh-fMRI in the same subgroup of patients (*r* = 0.79 ± 0.21).

In this study in most cases rs-fMRI seemed to be sensitive to detect impaired CVR in patients with MMA. Our results suggest that rs-fMRI might have potential in becoming a routine clinical examination after further research [42, 43]. A major advantage of rs-fMRI over externally hypercapnia-triggered fMRI is that the non-invasive procedure requires no complex inhalation and monitoring equipment and minimal patient cooperation. In bh-fMRI examinations, problems could exist when patients have difficulties following precise respiratory instructions, which, for example, ensure that breath-hold periods are performed after expiration [11, 41]. Therefore, rs-fMRI seems to be a widely available diagnostic method, which could be applied in a wide range of clinical examinations especially in patients who are not able to perform breath-hold tasks (e.g., pediatric patients or patients with cognitive impairment). In addition, rs-fMRI might be particularly helpful in estimating the CVR in severely ill patients in whom externally imposed hypercapnia, whether through breath-holding or through the inhalation of CO_2_-enriched gas, is undesirable. The rs-fMRI approach without manipulation of the CO_2_ concentration seems to be feasible in nearly all patients that are able to remain in the scanner for several minutes. The estimation of the CVR provides important information not only in patients with MMA [7, 10, 16, 40, 44, 45], but also in other diseases such as carotid artery stenosis [8, 25] and stroke [11, 28, 46].

During the rs-task the low-frequency changes of blood CO_2_ content attributed to natural variations in respiration are relatively small (2–4 mmHg) [24]. The reason why they seem to be sufficient for estimation of the CVR might be the sigmoidal relationship between the cerebral blood flow and the CO_2_ partial pressure [47–50]. Accordingly, the CVR, measured as %BOLD signal change/ΔCO_2_, is greater for small CO_2_ changes than for larger CO_2_ changes [50]. It has been shown that even subtle variations in respiratory rate and depth during natural breathing can be associated with significant BOLD signal changes [32, 33, 51]. However, in individual patients the BOLD signal changes induced by natural CO_2_ fluctuations could be too small to adequately estimate the CVR. This might be one reason why in some cases of this study only a low correlation between the rs-maps and the bh-maps could be measured.

In this study we used the frequency band-pass-filtered BOLD signal time course of the cerebellum as a surrogate of fluctuations in arterial CO_2_. Employing the cerebellum as a reference region in patients with MMA is a common approach [10, 21] because it is supplied by vertebrobasilar system that is typically unaffected by MMA and therefore represents a region where a physiological hemodynamic response is expected. Nevertheless, there is a risk that the cerebellar BOLD signal fluctuations in the frequency range selected in this study are not exclusively due to CO_2_ fluctuations, but are also influenced by other mechanisms such as neural activity, blood pressure fluctuations, or scanner noise [24, 33, 51, 52]. The selection of the frequency band-pass filter was based on the results of the study of Liu et al. [24] in which the end-tidal CO_2_ concentration correlated strongly with BOLD signal fluctuations within the frequency range between 0.02 and 0.04 Hz in healthy subjects. This might be another reason explaining the varying correlation coefficients of the individual patients in our study as it could be possible that this frequency band-pass filter is not equally appropriate in all patients. No doubt, real-time end-tidal measured CO_2_ might be a more precise regressor and it would enable absolute quantification of the CVR as %BOLD signal change/Δ end-tidal CO_2_, but it would be accompanied by complex CO_2_ monitoring and our aim was to investigate a procedure for the estimation of the CVR that is best available and easily feasible.

Another limitation of the simplified approach without absolute CVR quantification used in this study is that it does not allow inter-subject comparison of the CVR values across patients because the levels of the data ranges of the CVR values differ between individuals and a standardization has not yet been established. Further prospective studies might investigate how to increase the sensitivity of rs-fMRI and focus on open questions such as the best fitting reference time course for a voxel-wise cross correlation analysis and the necessary duration of the rs-scans to enable reliable results. In this study we used a relatively short acquisition time (5:12 min). In one patient this time period was not long enough to identify relevant differences between the signal time courses of regions with physiological and reduced CVR, because the differences were detectable only in the end of the acquisition time. Future studies could explore whether the sensitivity of rs-fMRI could be increased by using longer rs-tasks and analyzing exclusively time periods characterized by large variations in the spontaneous breathing pattern and in the BOLD signal time course. In this work, we compared a promising rs-fMRI approach [24, 28] with the diagnostic standard [^15^O]water PET and a bh-fMRI approach that has been proven to show good reproducibility [23] and very high agreement with [^15^O]water PET [10], and with which we also had good clinical experience. Nevertheless, there exists not yet a diagnostic standard of hypercapnia-triggered fMRI in Moyamoya diagnostics and other approaches exist [11, 12, 18, 31].

## Conclusions

In this work we demonstrated that rs-fMRI provides comparable evaluation of the CVR as bh-fMRI in patients with MMA. Especially in patients that are unable to cooperate fully, the rs-fMRI approach may be a helpful alternative to map CVR, e.g., when hypercapnic gas challenges are not feasible and patients are not able to perform breath-holding. Furthermore, rs-fMRI requires no additional equipment such as CO_2_ application and monitoring hardware as well as in room computer monitors to display breathing commands. The good agreement with bh-fMRI and [^15^O]water PET suggests that rs-fMRI is a promising method meriting further research.

## Data Availability

The data that support the findings of this study are available from the corresponding author, upon reasonable request.

## Abbreviations

ACZ: Acetazolamide
bh: Breath-hold
BOLD: Blood-oxygen-level-dependent
CPR: Cerebral perfusion reserve
CVR: Cerebrovascular reactivity
MMA: Moyamoya Angiopathy
rs: Resting-state

## Declarations

### Declarations

Preliminary results of the manuscript (comparison between rs-fMRI and bh-fMRI data sets in a subgroup of patients, no comparison between rs-fMRI and [15O]water PET) were presented at the 37th Annual Scientific Meeting of the European Society for Magnetic Resonance in Medicine and Biology (ESMRMB) (Zerweck L, Hauser TK, Roder C, Klose U. Measuring cerebrovascular reactivity in patients with Moyamoya Disease: Comparison of resting-state BOLD fMRI to breath-hold BOLD fMRI. Book of Abstracts. ESMRMB 2020 Online, 37th Annual Scientific Meeting 2020/09/30–2020/10/02. MAGMA. Oct 2020;33(Suppl 1):S48. https://doi.org/10.1007/s10334-020-00875-z) and the 55th Annual Conference of the German Society for Neuroradiology (Zerweck L, Hauser TK, Roder C, Ernemann U, Klose U. Hemodynamic evaluation of patients with Moyamoya: Comparison of resting-state-fMRI to breath-hold-fMRI. Book of Abstracts. neuroRAD 2020 Virtual 2020/10/06–08. Clinical Neuroradiology. 2020/06/01 2020;30(2):42–42. https://doi.org/10.1007/s00062-020-00951-w) and results will be presented at the 38th Annual Scientific Meeting of the European Society for Magnetic Resonance in Medicine and Biology (ESMRMB) and the 56th Annual Conference of the German Society for Neuroradiology.
